# Phytochemicals at the PPAR–NF‐*κ*B Interface: A Structured Narrative Review of Receptor Pharmacology, Inflammatory Signaling, and Translational Barriers

**DOI:** 10.1155/ppar/9577380

**Published:** 2026-10-29

**Authors:** Sabi Ur Rehman, Fazal Ur Rehman, Shafi Ullah Khan, Amir Zeb, Saleha Ashfaq, Shakir Ullah Khan, Waheed Ullah Hafiz

**Affiliations:** ^1^ Gomal Center of Pharmaceutical Sciences Faculty of Pharmacy Gomal University Dera Ismail Khan Pakistan gu.edu.pk; ^2^ Department of Pharmacy Forman Christian College (A Chartered University) Lahore Pakistan fccollege.edu.pk; ^3^ Independent Researcher Hérouville-Saint-Clair France; ^4^ Faculty of Pharmaceutical Sciences Abasyn University Peshawar Pakistan abasyn.edu.pk; ^5^ Department of Biology The University of Haripur Haripur Pakistan uoh.edu.pk; ^6^ Para-Clinic Department Faculty of Medicine Bayazid Rokhan University Kabul Afghanistan

**Keywords:** candidate selective PPAR modulators, chronic inflammation, NF-*κ*B, pharmacokinetics, phytochemicals, PPAR-*α*, PPAR-*γ*, transrepression

## Abstract

**Background:**

The peroxisome proliferator‐activated receptors (PPARs) and nuclear factor‐*κ*B (NF‐*κ*B) signaling axis serves as master molecular interface governing metabolic homeostasis and inflammatory resolution. Dysregulated reciprocal repression between PPAR‐*α*/*γ* and NF‐*κ*B contributes to chronic inflammatory diseases, metabolic syndrome, and malignancy. Synthetic full agonists (e.g., thiazolidinediones and fibrates) are associated with clinical liabilities including adipogenesis, fluid retention, hepatotoxicity, and cardiovascular adverse effects, underscoring the need for safer modulators.

**Objective and Rationale:**

This structured narrative review provides a rigorous mechanistic synthesis of plant derived phytochemicals with selective PPAR modulator (SPPARM) like pharmacology. We elucidate how distinct chemical classes may achieve targeted NF‐*κ*B transrepression with reduced transcriptional liability, summarize available ligand receptor binding data, and critically evaluate translational hurdles.

**Methods:**

A structured narrative review was conducted using a systematic style literature search across PubMed/MEDLINE, Scopus, and Web of Science from January 2000 through March 2026. In line with PRISMA 2020 principles for transparent reporting, original research articles were prioritized according to evidence type (clinical, preclinical, in vivo, and in vitro/in silico), with emphasis on pharmacodynamic metrics, binding kinetics (K_i_, EC_50_, and IC_50_), receptor dependency validation (knockout, antagonism), and pharmacokinetic data.

**Results:**

Chemically diverse phytochemicals including flavonoids, neolignans, stilbenoids and terpenoids exhibit partial or subtype selective PPAR agonism in reporter and binding assays. Mechanistically, many favor anti‐inflammatory transrepression over full coactivator‐dependent transactivation, with evidence for corepressor recruitment and inhibition of NF‐*κ*B nuclear translocation in selected models. However, integrated analysis reveals a persistent disconnect between submicromolar in vitro potency and negligible systemic exposure of free parent compound (low C_max_, extensive glucuronidation/sulfation), compounded by frequent reliance on unvalidated docking simulations and PPAR‐independent NF‐*κ*B inhibition.

**Conclusion:**

Phytochemicals with SPPARM‐like pharmacology represent a promising but unvalidated paradigm for nonsteroidal inflammation control. Realizing their potential will require next‐generation delivery systems (e.g., liposomes, nanoemulsions, and cyclodextrin complexes), mandatory genetic or pharmacological receptor validation, and biomarker‐driven clinical trials that document target engagement at achievable human exposure levels.

## 1. Introduction

Peroxisome proliferator‐activated receptors (PPARs) are ligand‐activated transcription factors of the nuclear receptor superfamily. They modulate vascular inflammation by negative crosstalk with transcription factors Nuclear Factor kappa B (NF‐*κ*B) and activator protein‐1 (AP‐1) [1]. PPARs have three isoforms: PPAR‐*α*, PPAR‐*γ*, and PPAR‐*β*/*δ*, each with distinct tissue distributions and physiological roles in energy metabolism and inflammation [2]. PPAR‐*β*/*δ* predominantly regulates fatty acid oxidation and energy expenditure in skeletal and cardiac muscles as well as modulates inflammatory signaling in metabolic diseases but its role in cancer and chronic inflammation is complex and sometimes protumorigenic and promotes tumor angiogenesis [3, 4]. By contrast PPAR‐*α* and PPAR‐*γ* are potent negative regulators of NF‐*κ*B driven inflammatory gene expression in vascular, hepatic, and adipose tissue acting through transrepression of p65, coregulator competition, and reinforcement of inhibitory checkpoints in the NF‐*κ*B cascade such as I*κ*B [5]. They primarily work by inhibiting NF‐*κ*B, a central transcription factor responsible for the expression of pro‐inflammatory cytokines like TNF‐*α*, IL‐6, IL‐1*β*, and adhesion molecules [6–8]. NF‐*κ*B is a transcription factor activated by immunological mediators in inflammation, is triggered in oxidative conditions, wherein reactive oxygen species (ROS) enhance the degradation of I*κ*B inhibitors, allowing NF‐*κ*B to translocate to the nucleus [9]. Upon activation by stimuli like lipopolysaccharides (LPS) or cytokines, I*κ*B is degraded, allowing the p65/p50 NF‐*κ*B complex to translocate to the nucleus and initiate transcription. PPARs inhibit NF‐*κ*B signals through transrepression preventing direct binding to DNA response regions [7, 10]. Consequently, the current review focused on PPAR‐*α* and PPAR‐*γ* as the principal isoforms mediating therapeutic PPAR‐NF‐*κ*B antagonism while acknowledging that PPAR‐*β*/*δ* represents an important but distinct topic beyond the current scope of this article.

PPARs regulate gene networks via two distinct mechanisms: canonical genomic transactivation and nongenomic transrepression [11, 12]. In canonical transactivation, ligand binding induces a conformational realignment of the C‐terminal ligand binding domain (LBD), specifically stabilizing the activation function‐2 (AF‐2) surface on Helix 12. This facilitates heterodimerization with the Retinoid X Receptor (RXR*α*, *β* or *γ*), triggering the dissociation of corepressors (such as NCoR and SMRT) and the recruitment of coactivator complexes including steroid receptor coactivators (SRC‐1/2/3), CREB‐binding protein (CBP)/p300, and PPAR‐*γ* coactivator‐1*α* (PGC‐1*α*). The resulting transcriptional complex binds sequence‐specific peroxisome proliferator response elements (PPREs; direct repeat‐1 [DR‐1] motifs: 5 ^′^‐AGGTCA‐N‐AGGTCA‐3 ^′^) to drive metabolic, adipogenic, and lipid‐oxidative gene programs [11].

Conversely, in anti‐inflammatory transrepression, PPARs negatively regulate pro‐inflammatory gene networks without direct PPRE binding [12]. Ligand binding triggers specific posttranslational modifications, predominantly ligand‐dependent SUMOylation of LBD such as SUMO‐1 conjugation at Lys365 on PPAR‐*γ* or Lys358 on PPAR‐*α* [13]. This SUMOylated receptor targets promoter‐bound NF‐*κ*B (p50/p65) or AP‐1 complexes and prevents the 19S proteasome‐dependent clearance of NCoR‐histone deacetylase 3 (HDAC3) complexes from pro‐inflammatory promoters, including TNF, IL6, and NOS2 maintaining them in a chromatin‐condensed state [12, 13]. Understanding this bifurcation between PPRE‐dependent transactivation and corepressor‐stabilizing transrepression provides the essential mechanistic foundation for evaluating selective PPAR modulators (SPPARM) [14].

### 1.1. Relevance to Metabolism and Inflammation

PPARs regulate genes involved in lipid metabolism and inflammation, and their activation is known to suppress pro‐inflammatory cytokine production [15]. In nonalcoholic fatty liver disease, hepatocyte‐specific PPAR‐*γ* disruption reduces hepatic steatosis but increases hepatic neutrophil infiltration following high‐fat diet feeding and binge ethanol exposure, emphasizing the complex role of PPAR‐*γ* in balancing metabolic and immune functions [16]. PPAR‐*γ* is abundantly expressed in adipose tissue, where it not only regulates adipogenesis but also suppresses inflammatory genes in macrophages (M1 to M2 polarization) [17]. Similarly, PPAR‐*α* in the liver regulates fatty acid oxidation and reduces inflammation [18]. Hypoxia induces inflammation independent of hypoxia‐inducible factor 1‐alpha by stimulating lipolysis, increasing free fatty acids, which downregulate the expression of PPAR‐*γ* and elevate the expression of macrophage inflammatory protein‐1*α* [19].

## 2. Methods

### 2.1. Literature Search Strategy

A structured and reproducible search was conducted across three primary electronic databases: PubMed/MEDLINE, Scopus, and Web of Science Core Collection, covering peer‐reviewed literature published from January 1, 2000, through March 31, 2026. The search strategy combined controlled vocabulary (Medical Subject Headings [MeSH] in PubMed) and free‐text title/abstract keywords utilizing Boolean logic (AND, OR).

The exact reproducible search strings developed for each database were structured as follows:

PubMed/MEDLINE: (“Peroxisome Proliferator‐Activated Receptors”[Mesh] OR “PPAR alpha” [tiab] OR “PPAR gamma”[tiab] OR “PPAR‐alpha”[tiab] OR “PPAR‐gamma”[tiab] OR “PPARa”[tiab] OR “PPARg”[tiab]) AND (“NF‐kappa B”[Mesh] OR “NF‐kappaB”[tiab] OR “NF‐*κ*B”[tiab] OR “transrepression”[tiab] OR “p65”[tiab] OR “RelA”[tiab]) AND (“Biological Products”[Mesh] OR “Phytochemicals”[Mesh] OR “Flavonoids”[Mesh] OR “phytochemical ^∗^”[tiab] OR “natural product ^∗^”[tiab] OR “polyphenol ^∗^”[tiab] OR “neolignan ^∗^”[tiab] OR “terpenoid ^∗^”[tiab] OR “sesquiterpene ^∗^”[tiab] OR “stilbenoid ^∗^”[tiab]) AND (“Inflammation”[Mesh] OR “Neoplasms”[Mesh] OR “Metabolic Diseases”[Mesh] OR “anti‐inflammatory”[tiab] OR “cancer”[tiab] OR “atherosclerosis”[tiab])).

Scopus: TITLE‐ABS‐KEY ((“PPAR‐alpha” OR “PPAR‐gamma” OR “PPAR‐a” OR “PPAR‐g”) AND (“NF‐kappaB” OR “NF‐*κ*B” OR “transrepression” OR “p65”) AND (“phytochemical ^∗^” OR “natural product ^∗^” OR “flavonoid ^∗^” OR “terpenoid ^∗^” OR “neolignan ^∗^” OR “stilbenoid ^∗^”) AND (“inflammation” OR “cancer” OR “atherosclerosis”)).

Web of Science (core collection): TS = ((“PPAR‐alpha” OR “PPAR‐gamma” OR “PPARa” OR “PPARg”) AND (“NF‐kappaB” OR “NF‐*κ*B” OR “transrepression” OR “p65”) AND (“phytochemical ^∗^” OR “natural product ^∗^” OR “flavonoid ^∗^” OR “terpenoid ^∗^” OR “neolignan ^∗^”) AND (“inflammation” OR “cancer” OR “metabolic”)).

In addition to database searching, secondary manual identification was executed via backward and forward citation chaining of key review papers and landmark experimental articles (*n* = 12).

### 2.2. Prospective Protocol Registration Statement and Bias Mitigation

As this manuscript is structured as a narrative review synthesized with systematic search methodology (PRISMA 2020 framework), a formal prospective protocol was not registered in public registries such as PROSPERO or INPLASY, which prioritize clinical interventional systematic reviews. Titles, abstracts, and eligible full texts were screened by the review team using the predefined criteria, with disagreements resolved through discussion and consensus.

### 2.3. Inclusion and Exclusion Criteria

Studies were systematically screened using the following predefined criteria:

**Inclusion criteria**

1. Peer reviewed original research reporting PPAR‐*α*/*γ* activation coupled with downstream NF‐*κ*B modulation.
2. Pharmacological evaluation of pure, chemically isolated phytochemicals, or well characterized bio fractions.
3. Studies utilizing validated in vitro (cell‐based receptor assays, macrophages/endothelial models), in vivo (rodent models of inflammation, metabolic syndrome, or carcinogenesis) or clinical models of inflammatory, neoplastic or metabolic diseases.
4. Structural, biophysical, or computational studies (molecular docking, molecular dynamics, SPR, TR‐FRET, and crystallographic analysis) detailing ligand LBD amino acid interactions.
5. Full text articles published in English language.

**Exclusion criteria**

1. Uncharacterized or unstandardized crude plant extracts where pharmacological activity cannot be attributed to a specific phytochemical entity.
2. Studies solely focus on the PPAR‐*β*/*δ* isoforms without cross evaluation of PPAR‐*α*/*γ* because of divergent and controversial oncogenic or angiogenic profiles.
3. Studies evaluating generic antioxidant effects lacking mechanistic insights on PPAR–NF‐*κ*B molecular signaling axis.
4. Nonoriginal records, including conference abstracts, editorials, book chapters, retracted articles, or nonpeer‐reviewed preprints.

### 2.4. Study Selection, Data Extraction and Evidence Stratification

Search records were exported to reference management software, and duplicate records (*n* = 123) were electronically removed. Two independent authors screened the remaining 291 titles and abstracts. Full text articles (*n* = 80) were assessed against the inclusion and exclusion criteria, and any discrepancies were resolved through discussion and consensus with a third author. After deduplication across the database and citation‐search pathways, 40 unique primary studies met the inclusion criteria and were included in the qualitative synthesis (Figure 1).

**Figure 1:**
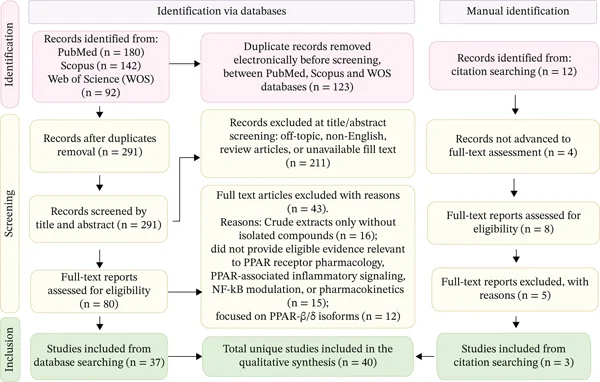
Literature search and study‐selection flow diagram. Records were identified through PubMed, Scopus, Web of Science, and manual citation searching. After duplicate removal, title/abstract screening, and full‐text eligibility assessment, 40 unique primary studies were included in the qualitative synthesis. The search and selection process was reported in accordance with PRISMA 2020 reporting principles and was used to inform this structured narrative review.

From each eligible study, extracted data included botanical source, phytochemical identity, chemical class, PPAR isoform targeted, quantitative potency metrics (K_i_, EC_50_, and IC_50_), binding pocket residues, target validation method, and downstream NF‐*κ*B modulation.

To prevent overinterpretation of preliminary computational data and clearly distinguish clinical efficacy from cell‐free assays, all extracted evidence was stratified into three hierarchical tiers:

• Level 1 (clinical evidence): Randomized controlled trials (RCTs), open‐label human studies, and clinical pharmacokinetic evaluations.
• Level 2 (preclinical in vivo evidence): Established animal disease models (e.g., DSS‐induced colitis, LPS‐induced endotoxemia, high‐fat diet atherosclerosis/NAFLD, tumor xenograft models) incorporating functional target engagement markers.
• Level 3 (mechanistic in vitro/in silico evidence): Cell‐culture models (e.g., RAW 264.7, THP‐1, and HepG2), transactivation luciferase reporter assays, coimmunoprecipitation, surface plasmon resonance (SPR), and molecular docking/dynamics simulations.

## 3. Structural Biology of PPAR′ LBD and Selective Modulation

### 3.1. Subtype Specific Architecture of Ligand Binding Pocket

The LBD; E/F domain of PPARs comprises a canonical three layered *α*‐helical sandwich of 13 *α*‐helices and a four‐stranded *β*‐sheet enclosing a substantial hydrophobic cavity (Figure 2) [11, 20]. Despite overall topological homology, distinct amino acid variations dictate subtype specific ligand recognition.

• PPAR‐*α* LBD: Encloses a binding pocket of approximately 1400 Å^3^ featuring a lipophilic and constrained entrance [21]. Polar coordination of carboxylate‐bearing ligands is mediated by a catalytic network comprising Ser280 (Helix 3), Tyr314 (Helix 5), His440 (Helix 10/11), and Tyr464 (Helix 12) [22]. The steric constraint imposed by Tyr314 (corresponding to the less bulky His323 in PPAR‐*γ)* enforces selectivity for linear, lipophilic fatty acids, and fibrate pharmacophores while excluding bulkier natural scaffolds [21, 22]. Furthermore, high‐potency PPAR‐*α* coordination involves Arm III interactions, where agonists form hydrogen bonds with Thr279 to stabilize Helix 3 [21].
• PPAR‐*γ* LBD: PPAR‐*γ* LBD comprises a large Y‐shaped binding cavity of 1300–1400 Å^3^ divided into three distinct sub arms (Arms I, II, and III) accessed via an extended, flexible *Ω*‐loop. Synthetic full agonists (such as thiazolidinediones) occupy Arm I, establishing a quadripartite hydrogen‐bonding network with Ser289 (Helix 3), His323 (Helix 5), His449 (Helix 10/11), and Tyr473 (Helix 12/AF‐2) [23, 24]. Direct anchoring to Tyr473 firmly locks Helix 12 in the active “closed” conformation, assembling the coactivator‐binding charge clamp required for robust transcriptional transactivation of adipogenic gene programs [14, 23].

**Figure 2:**
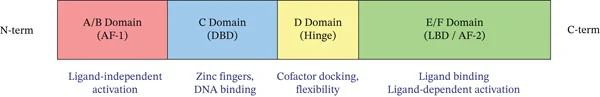
Structural architecture and functional domains of PPARs. Schematic representation of the canonical nuclear‐receptor domain organization of PPARs. The N‐terminal A/B domain contains the ligand‐independent activation function‐1 (AF‐1); the C domain contains the DNA‐binding domain (DBD) with zinc‐finger motifs; the flexible D domain represents the hinge region and contributes to receptor conformational flexibility and protein–cofactor interactions; and the C‐terminal E/F domain contains the LBD and ligand‐dependent activation function‐2 (AF‐2). The figure is schematic and not drawn to scale.

### 3.2. Structural Mechanism of Phytochemical Derived SPPARMs

Unlike synthetic full agonists, some plant derived bioactive compounds (e.g., neolignans like honokiol and magnolol, flavonoids like apigenin and quercetin, and amorfrutins) may display partial or context dependent PPAR pharmacology reminiscent of SPPARMs, but most lack the comprehensive receptor pharmacology evidence required for classification as validated SPPARMs (Figure 3 and Table 1) [14, 27]. Structural and molecular docking analyses demonstrate that natural ligands frequently bypass direct hydrogen bonding with Tyr473 on Helix 12 [14, 23]. Instead, these compounds primarily occupy Arm II and Arm III or anchor into the hydrophobic *β*‐sheet pocket, coordinating with residues such as Cys285, Arg288, Ile341, and Met364 [14, 27]. Concurrently, this alternative binding mode induces discrete allosteric transitions that preserve the receptor′s ability to undergo ligand‐dependent SUMOylation, stabilize NCoR/SMRT corepressor complexes, and physically transrepress NF‐*κ*B (p50/p65) promoter activity [12, 13].

**Figure 3:**
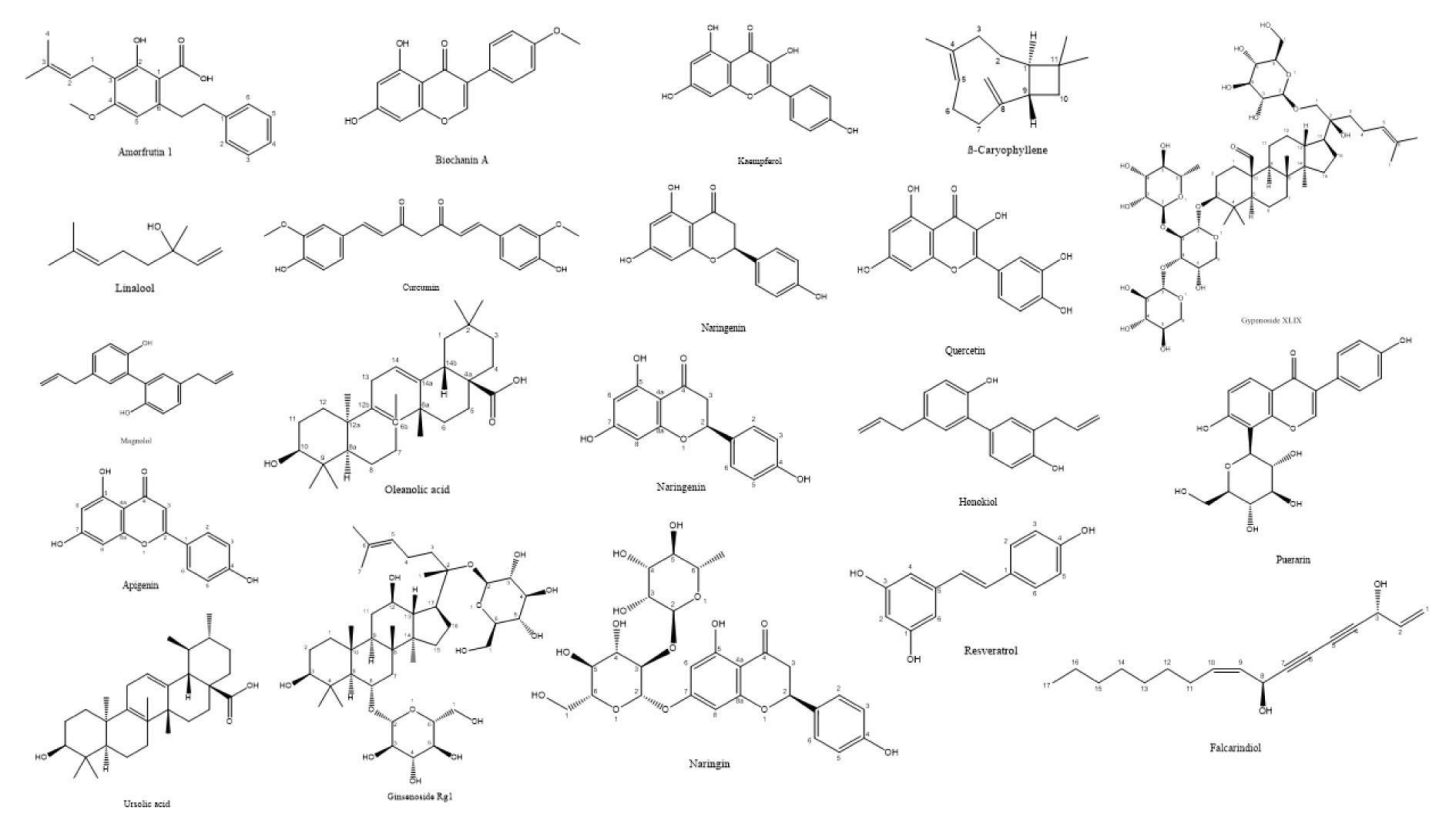
Chemical structures of representative phytochemicals investigated in relation to the PPAR–NF‐*κ*B signaling axis. The structures represent chemically diverse compounds—including flavonoids, neolignans, stilbenoids, curcuminoids, terpenoids, and triterpenoid saponins—that have been evaluated for direct PPAR binding, receptor‐dependent activity, PPAR‐associated signaling, NF‐*κ*B modulation, or translational pharmacokinetic relevance. The available evidence is heterogeneous; compounds shown are not uniformly validated direct PPAR ligands or proven PPAR‐mediated NF‐*κ*B transrepressors.

**Table 1:** Quantitative receptor pharmacology of representative phytochemical PPAR modulators supported by primary experimental evidence.

Phytochemical	Chemical class and representative natural source(s)	PPAR isoform	Quantitative pharmacology	Experimental validation	Evidence level	Primary source
Amorfrutin 1	Isoprenoid‐substituted benzoic acid, *Amorpha fruticosa* and *Glycyrrhiza foetida*	Preferential PPAR‐*γ* partial agonist	PPAR‐*α* *K*_*i*_ = 27 * μ*M; PPAR‐*β*/*δ* *K*_*i*_ = 27 * μ*M; PPAR‐*γ* *K*_*i*_ = 0.236 * μ*M; EC_50_ = 0.458 * μ*M; efficacy 39% vs rosiglitazone	Competitive TR‐FRET binding, reporter assay, cofactor recruitment, x‐ray cocrystal structure, PPAR‐*γ* knockdown and diet induced obese or db/db mouse studies	Level 2	[25]
Amorfrutin 2	Isoprenoid‐substituted benzoic acid, *Amorpha fruticosa* and *Glycyrrhiza foetida*	Preferential PPAR‐*γ* partial agonist	PPAR‐*α* *K*_*i*_ = 25 * μ*M; PPAR‐*β*/*δ* *K*_*i*_ = 17 * μ*M; PPAR‐*γ* *K*_*i*_ = 0.287 * μ*M; EC_50_ = 1.200 * μ*M; efficacy 30%	Competitive TR‐FRET binding and PPAR‐*γ* reporter assay	Level 3	[25]
Amorfrutin 3	Isoprenoid‐substituted benzoic acid, *Amorpha fruticosa* and *Glycyrrhiza foetida*	Preferential PPAR‐*γ* partial agonist	PPAR‐*α* *K*_*i*_ = 115 * μ*M; PPAR‐*β*/*δ* *K*_*i*_ = 68 * μ*M; PPAR‐*γ K_i_ =* 0.352 *μ*M; EC_50_ = 4.500 *μ*M; efficacy 22%	Competitive TR‐FRET binding and PPAR‐*γ* reporter assay	Level 3	[25]
Amorfrutin 4	Isoprenoid‐substituted benzoic acid, *Amorpha fruticosa* and *Glycyrrhiza foetida*	Preferential PPAR‐*γ* partial agonist	PPAR‐*α* *K*_*i*_ = 8 * μ*M; PPAR‐*β*/*δ* *K*_*i*_ = 6 * μ*M; PPAR‐*γ* *K*_*i*_ = 0.278 * μ*M; EC_50_ = 0.979 * μ*M; efficacy 15%	Competitive TR‐FRET binding and PPAR‐*γ* reporter assay	Level 3	[25]
Falcarindiol	Polyacetylene; *Notopterygium incisum*	Selective partial PPAR‐*γ* agonist in the reported reporter system, no PPAR‐*α* or PPAR‐*β*/*δ* activation reported	PPAR‐*γ* *K*_*i*_ = 3.07 * μ*M; and EC_50_ = 3.29 * μ*M; maximal activation 3.26‐fold versus vehicle, compared with 9.28‐fold for pioglitazone	Purified human PPAR‐*γ* LBD competitive TR‐FRET binding; HEK‐293 PPAR‐*γ* reporter assay; activity blocked by T0070907; 3 T3‐L1 adipogenesis/glucose uptake experiment	Level 3	[26]
Honokiol	Neolignan; *Magnolia officinalis*	PPAR‐*γ* preferring partial agonist; PPAR‐*α* and PPAR‐*β*/*δ* binding reported at three to four fold higher concentrations	PPAR‐*γ* *K*_*i*_ = 22.86 * μ*M; EC_50_ = 3.9 * μ*M; maximum activation approximately 2‐fold, compared with approximately 12‐fold for pioglitazone	Purified human PPAR‐*γ* LBD binding, reporter assay, 3 T3‐L1 and mouse embryonic fibroblast adipogenesis models; KKAy mouse study	Level 2	[27]
Gypenoside XLIX	Dammarane type triterpene saponin; *Gynostemma pentaphyllum*	Selective PPAR‐*α* activator in the reported transactivation assay; no activity against PPAR‐*γ* or PPAR‐*β*/*δ* reported	PPAR‐*α* reporter EC_50_ = 10.1 * μ*M	PPAR‐*α* reporter assay; reduction of LPS and TNF‐*α* stimulated NF‐*κ*B reporter activity; MK‐886 reversed the inhibitory effect	Level 3	[28, 29]

*Note:* Chemical classifications and selected botanical sources are provided for compound identification and pharmacognostic context. Representative natural occurrence was compiled from authoritative surveys of PPAR‐active natural products and related phytochemicals [14, 30, 31]. The listed source does not indicate the plant material, plant part, extraction method, compound purity, formulation, or commercial supplier used in the cited pharmacological study. For widely distributed phytochemicals, representative rather than exhaustive botanical sources are reported. *K_i_* values were obtained using competitive TR‐FRET ligand binding assays for the PPAR isoforms indicated. EC_50_ and efficacy values for amorfrutins were determined only in the PPAR‐*γ* reporter gene assay; therefore PPAR‐*α* and PPAR‐*β*/*δ* reporter EC_50_ values are reported in the cited study. The amorfrutin efficacy percentage is the maximum PPAR*γ* reporter response relative to rosiglitazone in the original experimental system. Evidence level denotes the highest level of evidence assessed in this review: Level 1, human clinical or clinical pharmacokinetic data; Level 2, in vivo preclinical evidence; and Level 3, biochemical, cellular, reporter‐based, structural, or computational evidence. Values generated using different assay platforms must not be compared as interchangeable measures of potency.

## 4. Multilayered Mechanisms of PPAR‐NF‐*κ*B Antagonism

### 4.1. Canonical PPAR–NF‐**κ**B Transrepression

PPAR‐mediated antagonism of NF‐*κ*B occurs principally through transrepression, an anti‐inflammatory mechanism that does not necessarily require direct binding of PPARs to PPREs. The principal receptor‐mediated and upstream signaling events involved in this regulatory axis are summarized in Figure 4. Ligand‐activated PPAR‐*α* and PPAR‐*γ* can suppress NF‐*κ*B‐dependent transcription through direct interaction with the RelA (p65) subunit, competition for limiting coactivators such as CBP/p300 and reinforcement of inhibitory checkpoints in the IKK–I*κ*B*α*–NF‐*κ*B cascade [7, 12].

**Figure 4:**
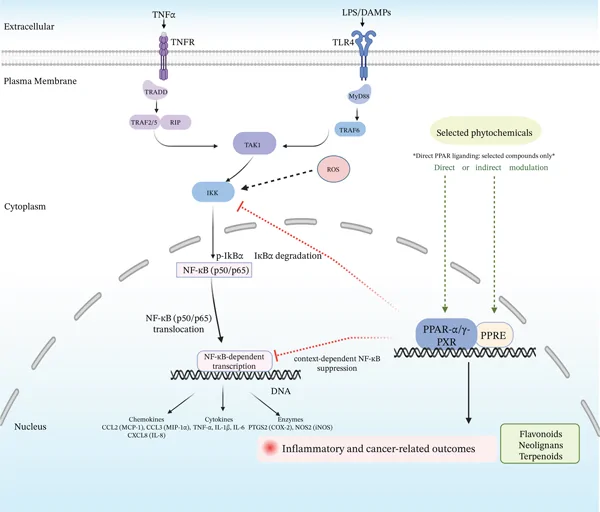
Conceptual model of PPAR–NF‐*κ*B crosstalk and potential phytochemical intervention points. Pro‐inflammatory stimuli, including TNF‐*α*/TNFR and TLR4‐associated signals, activate adaptor proteins and kinase cascades involving TRAF proteins, MyD88, TAK1, and IKK, promoting I*κ*B*α* phosphorylation, ubiquitination, and degradation and facilitating nuclear translocation of the p50/p65 NF‐*κ*B complex. NF‐*κ*B activation induces inflammatory cytokines, chemokines, and enzymes, including TNF‐*α*, IL‐1*β*, IL‐6, CCL2/MCP‐1, CCL3/MIP‐1*α*, CXCL8/IL‐8, PTGS2/COX‐2, and NOS2/iNOS. Selected phytochemicals may influence this axis through direct PPAR ligand activity, indirect modulation of upstream signaling, or PPAR‐independent effects. Ligand‐activated PPAR‐*α* and PPAR‐*γ* can suppress NF‐*κ*B‐dependent transcription through context‐dependent mechanisms, including cofactor competition, interference with p65/RelA activity, I*κ*B*α* regulation, and stabilization of promoter‐associated corepressor complexes. The PPRE represents canonical PPAR transcriptional activity and does not imply direct PPRE activation by every phytochemical shown. Accordingly, this figure is a mechanistic framework rather than a universal causal model. Abbreviations: TNFR, tumor necrosis factor receptor; TLR4, toll‐like receptor 4; DAMPs, damage‐associated molecular patterns; MyD88, myeloid differentiation primary response 88; TRAF, TNF receptor‐associated factor; TAK1, transforming growth factor‐*β*‐activated kinase 1; RIP, receptor‐interacting protein; TRADD, TNFR‐associated death domain; IKK, I*κ*B kinase; I*κ*B*α*, inhibitor of *κ*B*α*; NF‐*κ*B, nuclear factor‐*κ*B; PPAR, peroxisome proliferator‐activated receptor; RXR, retinoid X receptor; PPRE, PPAR response element; ROS, reactive oxygen species. Created with http://BioRender.com.

PPAR activation may reduce IKK‐dependent I*κ*B*α* phosphorylation or promote I*κ*B*α* expression and stability, thereby limiting nuclear accumulation of the p50/p65 complex and reducing transcription of inflammatory genes, including TNF, IL1B, IL6, CCL2, PTGS2/COX2, and NOS2/iNOS [7, 8]. Nevertheless, decreased NF‐*κ*B activity following phytochemical exposure does not, by itself, establish PPAR‐dependent transrepression, because phytochemicals may also inhibit inflammatory kinases, oxidative‐stress signaling, or NF‐*κ*B directly through PPAR‐independent mechanisms [10, 12].

### 4.2. Direct and Indirect PPAR Modulation by Phytochemicals

A central consideration in phytochemical pharmacology is whether a compound directly engages the PPAR LBD or instead alters PPAR expression, posttranslational or downstream activity through upstream signaling pathways. Direct PPAR modulation should be assigned only when purified compounds are supported by receptor transactivation, binding or cofactor‐recruitment assays, and preferably by pharmacological or genetic receptor‐dependency experiments [14, 30]. Natural neolignans such as honokiol have experimental evidence of PPAR‐*γ* LBD and partial agonism in PPAR‐*γ* dependent luciferase reporter assay. In contrast, for many other phytochemicals, evidence of direct LBD binding remains limited or is based principally on computational docking; such predictions should be presented as mechanistic hypotheses rather than definitive evidence of receptor agonism [14, 27].

Many phytochemicals also influence the PPAR–NF‐*κ*B axis indirectly through AMPK, SIRT1, MAPK, PI3K/Akt, and redox‐sensitive pathways. The AMPK–SIRT1 axis can modulate PPAR‐*γ* transcriptional programs through deacetylation of PPAR‐*γ* and regulation of PGC‐1*α*, whereas inhibition of TAK1, p38, JNK, ERK, or PI3K/Akt may reduce IKK activation and preserve I*κ*B*α* independently of direct PPAR liganding [32, 33]. Accordingly, increased PPAR expression or reduced NF‐*κ*B activity after phytochemical treatment should not alone be interpreted as proof of direct receptor activation. Definitive assignment of PPAR‐dependent anti‐inflammatory activity requires complementary binding or transactivation data and antagonist‐, gene‐silencing‐, or knockout‐based validation [14, 34].

### 4.3. PPAR‐Independent Suppression of NF‐*κ*B by Phytochemicals

Phytochemicals commonly act through multiple molecular targets; therefore, their suppression of NF‐*κ*B may occur independently of PPAR activation. This consideration is particularly relevant for compounds that simultaneously affect oxidative stress, TLR4 signaling, kinase cascades, and inflammatory transcription factors [10].

Certain electrophilic natural products, particularly sesquiterpene lactones, can interfere directly with the IKK‐I I*κ*B*α*‐NF‐*κ*B signalling cascade. Parthenolide has been reported to inhibit TNF‐*α* induced IKK activity, thereby reducing I*κ*B*α* degradation and nuclear NF‐*κ*B activation [35, 36]. Natural products may additionally suppress NF‐*κ*B signaling through redox modulation, attenuation of intracellular ROS, or inhibition of TLR4‐, MAPK‐, and PI3K/Akt‐dependent upstream pathways. These convergent anti‐inflammatory actions do not by themselves establish direct PPAR engagement or PPAR‐dependent transrepression [10, 32]. Polyphenols can further attenuate inflammatory signaling by reducing intracellular ROS and modulating upstream TLR4‐, MAPK‐, and PI3K/Akt‐dependent pathways. Although these actions may converge on lower NF‐*κ*B activity, antioxidant or kinase‐inhibitory effects alone do not demonstrate PPAR engagement [10, 32].

Therefore, receptor dependency should be tested using selective antagonists, gene silencing, CRISPR‐based editing, or receptor‐knockout models. Without these controls, the appropriate conclusion is that a phytochemical is associated with both PPAR modulation and NF‐*κ*B inhibition, rather than proven PPAR‐mediated transrepression [12, 14].

### 4.4. Epigenetic Regulation and Posttranslational Modifications

PPAR‐mediated transrepression is influenced by posttranslational modifications and promoter‐associated corepressor complexes. One of the best‐characterized mechanisms involves ligand‐dependent SUMOylation of PPAR**-*γ*** which promotes its association with NCoR‐HDAC3 complexes at selected inflammatory promoters [13]. In this model, SUMOylated PPAR‐***γ*** prevents the signal‐dependent, ubiquitin/19S‐proteasome‐mediated clearance of NCoR‐containing complexes. Retention of the NCoR–HDAC3 complex sustains transcriptional repression and limits NF‐*κ*B‐dependent expression of selected inflammatory genes [37]. PPAR‐*α* is also subject to SUMOylation‐dependent corepressor regulation. In human PPAR‐*α*, SUMO‐1 modification at Lys185 has been reported to promote selective recruitment of NCoR, rather than SMRT, and to suppress the transcriptional activity of a subset of PPAR‐*α* target genes [38]. Other PPAR posttranslational modifications, including phosphorylation, acetylation, ubiquitination, and O‐GlcNAcylation can alter receptor stability, localization, cofactor recruitment, and transcriptional output in an isoform‐, ligand‐, and cell‐context‐dependent manner [18].

In addition, noncoding RNAs regulate inflammatory pathways relevant to PPAR‐NF‐*κ*B crosstalk. For example, miR‐146a and miR‐223 can restrain inflammatory signaling, whereas miR‐155 often promotes inflammatory activation; these miRNAs regulate mediators such as IRAK1, TRAF6, macrophage polarization, and NF‐*κ*B pathway activity [39].

### 4.5. Transcriptional Crosstalk Beyond NF‐*κ*B

PPAR‐mediated anti‐inflammatory actions are embedded within a broader transcriptional network involving AP‐1, STAT proteins, Nrf2, FOXO factors, and HIF‐1*α*. These interactions can influence inflammation, oxidative‐stress adaptation, metabolic reprogramming, cell survival, and angiogenesis, although their direction and functional consequences depend on the PPAR isoform, ligand, cell type, and disease context [12, 40]. PPAR activation can antagonize AP‐1‐ and STAT‐dependent inflammatory transcription through competition for limiting transcriptional coactivators, altered kinase signaling, and context‐dependent protein–protein interactions. It is more accurate to describe these effects as transcriptional and signaling crosstalk rather than assuming a direct inhibitory interaction in every system [7, 12].

PPAR and Nrf2 pathways may cooperate to limit oxidative and inflammatory injury. Nrf2 drives antioxidant‐response‐element‐dependent cytoprotective genes, whereas PPARs regulate lipid metabolism and anti‐inflammatory transcriptional programs; their convergence may decrease oxidative stress‐associated NF‐*κ*B activation [34, 41].

PPAR signaling can also intersect with FOXO‐ and HIF‐1*α*‐regulated programs involved in energy metabolism, oxidative‐stress responses, and hypoxic adaptation. However, this evidence is strongly dependent on the experimental model; these pathways should therefore be interpreted as relevant mechanistic context and priorities for future validation, rather than universal consequences of every phytochemical PPAR modulator [40].

## 5. Pharmacological Characterization and Stratified Evidence Synthesis

### 5.1. Framework for Evidence Appraisal and Candidate SPPARM Classification

The pharmacological evidence linking phytochemicals to the PPAR‐NF‐*κ*B axis is heterogeneous and must be interpreted according to the type of supporting experiment. Direct ligand engagement is supported by experiments such as competitive binding to purified PPAR LBD, cofactor‐recruitment assays, receptor‐transactivation reporter assays, or structural studies. In contrast, changes in PPAR mRNA or protein abundance, favorable docking scores, and concurrent suppression of NF‐*κ*B do not independently establish direct receptor binding or PPAR‐dependent transrepression [14, 30]. Representative chemical scaffolds of the phytochemicals discussed in this section are shown in Figure 3.

In this review, the term candidate SPPARM is reserved for purified compounds that demonstrate direct PPAR binding and/or receptor‐dependent functional activation together with partial or selective transactivation relative to a full synthetic agonist in the same experimental system. Compounds supported only by molecular docking, altered receptor expression, or broad anti‐inflammatory activity are instead described as PPAR‐associated or preliminary modulators. This distinction is important because many phytochemicals are polypharmacological and may suppress NF‐*κ*B through PPAR‐independent mechanisms [10, 25, 27].

Evidence was stratified into three levels. Level 1 denotes human clinical or clinical pharmacokinetic evidence; Level 2 denotes in vivo preclinical evidence from disease‐relevant animal models; and Level 3 denotes biochemical, cellular, reporter‐based, structural, or computational evidence. The assigned level represents the highest form of evidence available for the compound; it does not necessarily indicate that PPAR‐dependent NF‐*κ*B transrepression has been demonstrated at that same level.

To distinguish well‐characterized receptor pharmacology from preliminary associations, Table 1 presents phytochemicals for which quantitative receptor data are available from primary experiments. Table 2 summarizes additional compounds relevant to the PPAR–NF‐*κ*B axis and explicitly identifies the evidentiary limitation that prevents their classification as validated direct SPPARMs.

**Table 2:** Supporting phytochemicals reported to influence the PPAR‐NF‐*κ*B axis: evidence status and interpretation.

Phytochemical; chemical class; representative source(s)	Compound specific primary evidence	Highest evidence level	Interpretation	Key limitation	Primary reference
Curcumin (curcuminoid; *Curcuma longa*)	In primary rat cortical neurons exposed to oxygen–glucose deprivation/reoxygenation, curcumin increased PPAR‐*γ* expression and transcriptional activity, reduced IKK phosphorylation, I*κ*B degradation, reduced NF‐*κ*B nuclear translocation and promoted PPAR‐*γ*–p65 interaction. These effects were attenuated by GW9662 or PPAR‐*γ* siRNA.	Level 3	Functional PPAR‐*γ* associated inhibition of NF‐*κ*B in a neuronal injury model.	Evidence is cellular and model specific; this does not establish direct LBD binding, quantitative receptor affinity or clinical target engagement.	[42]
Quercetin (flavanol; *Allium cepa*, *Camellia sinensis*)	In myocardial ischemia/reperfusion models, quercetin increased PPAR‐*γ* expression, reduced NF‐*κ*B activation, and attenuated cardiac injury. GW9662 or PPAR‐*γ* siRNA partially reduced these protective and NF‐*κ*B inhibitory effects. In LPS‐treated human gingival fibroblasts, GW9662 reversed quercetin‐associated suppression of inflammatory cytokines and NF‐*κ*B signaling.	Level 2	Preclinical PPAR‐*γ* linked inhibitor of NF‐*κ*B signaling with pharmacological and gene silencing support.	Direct purified LBD binding, quantitative affinity, and comprehensive PPAR isoform selectivity remain insufficiently characterized.	[43, 44]
Resveratrol (stilbenoid; *Vitis vinifera*)	In PMA differentiated THP‐1 macrophages, resveratrol activated a PPAR‐*γ*, reduced EMMPRIN expression and MMP‐9 activity, whereas GW9662 attenuated the inhibitory effects of resveratrol on EMMPRIN and MMP‐9.	Level 3	Multitarget phytochemical with separable PPAR‐*γ* dependent and PPAR‐*γ* independent actions.	This study does not directly establish PPAR‐*γ* dependent NF‐*κ*B transrepression; resveratrol is a multitarget compound with additional PPAR‐independent actions.	[45]
Thymoquinone (monoterpenoid; *Nigella sativa*)	In dextran‐sulfate‐sodium‐induced colitis, thymoquinone increased colonic epithelial PPAR‐*γ* expression and reduced inflammatory responses and NF‐*κ*B associated signaling.	Level 2	PPAR associated preclinical anti‐inflammatory candidate.	Increased PPAR‐*γ* expression is not direct evidence of LBD binding; receptor dependency was not established by antagonist or genetic experiments in this model.	[46]
Magnolol (neolignan; *Magnolia officinalis*)	In LPS induced acute lung injury in rats, magnolol reduced inflammatory cytokines, increased PPAR‐*γ*, suppressed NF‐*κ*B activation, and its protective effects were attenuated by GW9662.	Level 2	Preclinical evidence supports partly PPAR‐*γ* dependent NF‐*κ*B suppression in acute lung injury.	The evidence is disease model specific and does not provide purified LBD affinity or comprehensive isoform selectivity data.	[47]
4‐O‐methylhonokiol (neolignan; *Magnolia officinalis*)	In human prostate cancer cells, 4‐O‐methylhonokiol activated PPAR‐*γ*, induced p21, suppressed NF‐*κ*B activity and inhibited tumor growth in xenograft experiments.	Level 2	PPAR‐*γ* linked suppression of NF‐*κ*B in a cancer context.	Cancer specific evidence should not be generalized to all inflammatory diseases or to the parent compound honokiol.	[48]
Apigenin (flavanol; *Matricaria chamomilla*)	In macrophage and diet‐induced‐obesity models, apigenin modulate PPAR‐*γ* activity, reduce p65 nuclear translocation and NF‐*κ*B activity, promote M2 macrophage polarization, and attenuate obesity associated inflammation.	Level 2	Preclinical PPAR‐*γ* modulator/activator with anti‐inflammatory activity.	Quantitative binding affinity, complete subtype selectivity characterization and independent receptor dependency validation across disease model remain limited.	[49]
Biochanin A (isoflavone; *Trifolium pratense*)	In LPS‐stimulated macrophages, biochanin A reduced TNF‐*α* and IL‐6 secretion; GW9662 significantly attenuated the anti‐inflammatory effects.	Level 3	Functional PPAR‐*γ* associated anti‐inflammatory candidate.	The study does not establish purified receptor binding, quantitative affinity, or complete isoform selectivity.	[50]
Ginsenoside Rg1 (dammarane‐type triterpene saponin; *Panax ginseng*)	In manganese‐induced hepatotoxicity experiments, ginsenoside Rg1 increased PPAR‐*γ* expression and attenuated TLR4/MyD88/MAPK‐associated inflammatory signaling and cytokine production.	Level 2	Preclinical candidate, associated with modulation of PPAR‐*γ* signaling and inflammatory pathways.	The study does not establish direct PPAR‐*γ* LBD binding or fully separate PPAR‐*γ* dependent effects from upstream TLR4/MyD88/MAPK regulation.	[51]
Morin hydrate (flavonol; *Morus alba*)	In experimental colitis, Morin hydrate was associated with enhanced PPAR‐*γ* expression/promoter activity, reduced inflammatory mediators and NF‐*κ*B phosphorylation and activation of Nrf2 associated antioxidant signaling.	Level 2	Multi target phytochemical with PPAR‐*γ*/Nrf2 associated activity in preclinical colitis models.	Docking, expression, and promoter‐activity findings do not independently establish direct PPAR‐*γ*‐LBD binding or exclusive PPAR dependent activity.	[52]
Methoxyeugenol (phenylpropanoid; *Myristica fragrans*)	In hepatic stellate cell and experimental liver fibrosis models, methoxyeugenol increased PPAR‐*γ* expression and attenuated NF‐*κ*B associated inflammatory and fibrotic response.	Level 2	PPAR‐*γ* associated anti‐fibrotic and anti‐inflammatory candidate.	Additional purified LBD binding, isoform selectivity, and independent receptor dependency evidence are required.	[53]

*Note:* Chemical classifications and selected botanical sources are provided for compound identification and pharmacognostic context. Representative natural occurrence was compiled from authoritative surveys of PPAR‐active natural products and related phytochemicals [14, 30, 31]. The listed source does not indicate the plant material, plant part, extraction method, compound purity, formulation, or commercial supplier used in the cited pharmacological study. For widely distributed phytochemicals, representative rather than exhaustive botanical sources are reported. “PPAR‐*γ*‐associated” denotes evidence of altered PPAR‐*γ* signaling together with anti‐inflammatory or NF‐*κ*B related outcomes; it does not itself prove direct ligand binding. The evidence level denotes the highest evidence type represented by the cited primary study: Level 1, human clinical or clinical‐pharmacokinetic evidence; Level 2, disease‐relevant in vivo preclinical evidence; and Level 3, biochemical, cellular, reporter‐based, structural, or computational evidence.

### 5.2. Phytochemicals With Quantitative Receptor Pharmacology

Amorfrutins are among the most comprehensively characterized plant‐derived selective PPAR‐*γ* modulators. In competitive TR‐FRET binding assays using purified PPAR‐*γ*, amorfrutins 1–4 exhibited *Ki* values of 0.236, 0.287, 0.352, and 0.278 *μ*M, respectively. In the corresponding reporter assay, their PPAR‐*γ* EC_50_ values were 0.458, 1.200, 4.500, and 0.979 *μ*M with maximal efficacies of 39%, 30%, 22%, and 15%, respectively, relative to rosiglitazone. The same work reported weaker binding to PPAR‐*α* and PPAR‐*β*/*δ* supporting preferential rather than absolute PPAR‐*γ* selectivity [25].

In addition to partial reporter activity, amorfrutin 1 was co‐crystallized with the PPAR‐*γ* LBD and shown to bind between Helix 3 and the *β*‐sheet with interactions involving Arg288 and Ser342. In diet‐induced obese and db/db mice, amorfrutin 1 improved insulin resistance and several metabolic and inflammatory parameters without the weight gain or hepatotoxicity observed with rosiglitazone in the reported models. These data provide a comparatively strong experimental basis for classifying amorfrutins as natural candidate SPPARMs, although human pharmacokinetic and clinical data remain unavailable [25].

Falcarindiol is a polyacetylene with experimentally demonstrated direct interaction with purified human PPAR‐*γ* LBD. It showed a *K_i_* of 3.07 *μ*M in a competitive TR‐FRET assay and activated a PPAR‐*γ* dependent luciferase reporter with an EC_50_ of 3.29 *μ*M. Its reporter activity was inhibited by the PPAR‐*γ* antagonist T0070907, and it activated PPAR‐*γ* without activating PPAR‐*α* or PPAR‐*β*/*δ* in the reported cellular reporter system. Falcarindiol therefore represents a useful direct‐binding, receptor‐antagonist‐validated example of a natural partial PPAR‐*γ* agonist, although its evidence remains predominantly in vitro [26].

Honokiol has also been characterized as a direct PPAR‐*γ* ligand. In the original study, honokiol bound purified human PPAR‐*γ* LBD with a *K_i_* of 22.86 *μ*M and activated a PPAR‐*γ* responsive luciferase reporter with an EC_50_ of 3.9 *μ*M. Compared with pioglitazone in the same reporter system, honokiol produced submaximal activation and did not induce marked adipogenic differentiation in 3T3‐L1 preadipocytes or mouse embryonic fibroblasts. In diabetic KKAy mice, oral honokiol reduced hyperglycemia and weight gain. These findings support classification of honokiol as a partial, nonadipogenic PPAR‐*γ* agonist in the reported systems; however, they do not demonstrate that all reported anti‐inflammatory or anticancer effects of honokiol are PPAR‐*γ* dependent [27].

Gypenoside XLIX provides a PPAR‐*α* oriented example that directly connects receptor modulation with NF‐*κ*B inhibition. It activated PPAR‐*α* in a reporter assay with a reported EC_50_ of 10.1 *μ*M while showing no activation of PPAR‐*γ* or PPAR‐*β*/*δ* in the corresponding assay. In macrophage and endothelial‐cell systems, gypenoside XLIX suppressed LPS‐ or TNF‐*α* induced NF‐*κ*B associated responses, and the PPAR‐*α* antagonist MK‐886 abolished the inhibition of stimulated NF‐*κ*B reporter activity. This is strong cellular evidence of PPAR‐*α* dependent NF‐*κ*B suppression, although it should be categorized as Level 3 unless an in vivo receptor‐dependency study is specifically incorporated [28, 29].

### 5.3. Interpretation of Supporting and Preliminary Evidence

Several widely studied phytochemicals, including curcumin, resveratrol, quercetin, thymoquinone, apigenin, biochanin A, and methoxyeugenol, have reported activity relevant to PPARs and NF‐*κ*B. However, they should not be treated as pharmacologically equivalent to direct‐binding partial agonists unless purified compound receptor assays and receptor‐dependency experiments are available in the relevant model. Their biological effects may reflect combinations of PPAR modulation, AMPK/SIRT1 regulation, TLR4/IKK/MAPK inhibition, redox effects, and other targets [10, 34].

Molecular docking can generate useful hypotheses about possible ligand‐LBD interactions, but it cannot independently establish *K_i_*, *K_D_*, agonist efficacy, subtype selectivity, coregulator recruitment, or receptor‐dependent cellular activity. This is especially relevant for PPARs because their ligand‐binding pockets are large and conformationally adaptable, and static docking procedures incompletely represent solvent effects, ligand‐induced Helix 12 dynamics, and coregulator‐associated conformational states. Therefore, docking‐only studies are classified as preliminary Level 3 evidence until supported by biochemical binding, reporter transactivation, cofactor‐recruitment, receptor‐antagonist, gene‐silencing, or knockout experiments [14, 54, 55].

Taken together, the available literature supports a small group of natural compounds as direct functional PPAR modulators, whereas most phytochemicals discussed in relation to the PPAR‐NF‐*κ*B axis remain PPAR‐associated or multitarget candidates. This evidence‐aware interpretation avoids overattributing NF‐*κ*B inhibition to PPAR transrepression and defines the experimental validation required for translational advancement.

### 5.4. Phytochemicals With Preliminary or Context Dependent Evidence

Additional phytochemicals identified during the structured literature search, including naringin/naringenin, kaempferol, ursolic acid, oleanolic acid, puerarin, *β*‐caryophyllene, and linalool, have reported effects on PPAR and/or NF‐*κ*B signaling. Their compound‐specific evidence, experimental models, receptor‐validation status, and principal limitations are summarized in Table S1. These phytochemicals are best regarded as preliminary or context‐dependent candidates rather than validated direct SPPARMs [43, 44].

## 6. Therapeutic Implications and Clinical Translation

### 6.1. Translational Barriers and Formulation Strategies

A central challenge in translating phytochemical PPAR–NF‐*κ*B modulators from experimental models to clinical use is the frequent disconnect between reported in vitro potency and pharmacologically relevant systemic exposure. This challenge is particularly relevant to compounds such as curcumin, thymoquinone, honokiol, and magnolol, which may have limited aqueous solubility, restricted absorption, extensive first‐pass metabolism, rapid clearance, or predominant conversion to conjugated metabolites. Consequently, cellular concentrations associated with PPAR modulation or NF‐*κ*B inhibition cannot be assumed to be attainable as free parent compound in systemic circulation or at the relevant disease site following conventional administration [56–58].

Curcumin provides a representative example. In an independent crossover pharmacokinetic study of nine healthy men, unconjugated curcumin concentrations remained below the lower limit of quantification of 2 nM after conventional curcumin preparations, high‐dose curcumin, curcumin administered with piperine, and Longvida. NovaSOL, a micellar lipid formulation, produced measurable unconjugated curcumin concentrations, with a median C_max_ of 14.9 nM (range, 6.7–38.4 nM) at 1 h; however, concentrations declined rapidly to a median of 1.2 nM by 4 h. Although total plasma curcuminoid concentrations were substantially higher after enzymatic hydrolysis of conjugates, these measurements do not represent free unconjugated curcumin exposure. The observed free curcumin concentrations remained at least approximately two orders of magnitude below the lower end of the 1–100 *μ*M range commonly used in in vitro experiments, with the precise exposure gap depending on the assay and cellular endpoint examined [57].

Formulation approaches, including lipid‐based systems, liposomes, nanoemulsions, polymeric micelles, phospholipid complexes, and cyclodextrin inclusion systems may improve solubilization, absorption, circulation time, or tissue delivery of selected phytochemicals. Nevertheless, exposure enhancement must be interpreted according to the formulation, species, dose, route, analyte measured, and comparator used. For example, a cationic liposomal thymoquinone formulation produced approximately 1.5 fold higher plasma concentrations and improved bioavailability relative to free thymoquinone in a preclinical pancreatic tumor model [59]. Similarly, lecithin‐based mixed polymeric micelles improved oral bioavailability of honokiol and magnolol in rats; however, the reported oral absolute bioavailability remained limited, at 4.8% for honokiol and 20.1% for magnolol in the optimized formulation [58].

However, formulation‐enhanced exposure alone does not establish therapeutic translation. Future studies should quantify parent compound and major metabolites, compare observed concentrations with receptor‐relevant concentrations from binding or reporter assays, and assess tissue distribution in disease‐relevant organs. Most importantly, improved pharmacokinetics should be linked to pharmacodynamic evidence of PPAR target engagement and NF‐*κ*B modulation, rather than inferred solely from symptomatic, metabolic, or nonspecific inflammatory outcomes. Selected quantitative pharmacokinetic parameters and formulation‐dependent exposure data for representative phytochemicals are summarized in Table S2.

### 6.2. Clinical Evidence, Safety, and Comparison With Synthetic Agonists

Human evidence for phytochemical modulation of the PPAR‐NF‐*κ*B axis remains limited, heterogeneous, and rarely accompanied by direct pharmacokinetic or target engagement measurements. Most clinical studies have evaluated metabolic indices, oxidative stress markers, circulating cytokines, or symptom‐based outcomes rather than simultaneous evidence of compound exposure, PPAR activation, and NF‐*κ*B suppression in the same participants. Therefore, improvements in glycemic control, lipid profile, C‐reactive protein, or other inflammatory biomarkers should not be interpreted as proof of PPAR‐dependent NF‐*κ*B transrepression. A summary of representative human studies with pathway relevant biomarkers or pharmacokinetic data is provided in Table 3.

**Table 3:** Human clinical and translational evidence for selected phytochemical PPAR modulators.

Phytochemical/formulation	Clinical population and study design	Dose and duration	Main human outcome	PPAR/NF‐*κ*B target‐engagement evidence	Key translational limitation	Primary reference
Resveratrol	Adults with Type 2 diabetes mellitus and coronary heart disease; randomized, double blind, placebo controlled trial; *n* = 56, 28 participants per group	500 mg/day for 4 weeks	Compared with placebo, resveratrol reduced fasting glucose, insulin, insulin resistance, total cholesterol/HDL‐cholesterol ratio, and malondialdehyde; it increased insulin sensitivity, HDL cholesterol, and total antioxidant capacity	PBMC *PPARG* and *SIRT1* expression increased significantly. No significant effect on measured inflammatory markers was reported	Short duration and small sample; PBMC gene expression does not establish direct receptor binding, tissue‐specific PPAR activation, or PPAR‐dependent NF‐*κ*B transrepression; no pharmacokinetic exposure assessment	[60]
Trans‐resveratrol	Adults with Type 2 diabetes mellitus; randomized, double blind, placebo controlled trial; *n* = 71	1000 mg/day for 8 weeks	Increased *p53* and *p21* mRNA expression and reduced the serum sCD163/sTWEAK ratio; no significant adjusted effect on serum sCD163 concentration	No significant change in PBMC *PPARA* or *p16* mRNA expression	A negative *PPARA* finding in PBMCs does not exclude effects in other tissues; NF‐*κ*B was not measured; no parent‐compound or metabolite pharmacokinetic assessment was performed	[61]
Resveratrol	Adults with nondialyzed stage 3–4 chronic kidney disease; randomized, double blind, placebo controlled crossover trial; *n* = 20; mean age 62.0 ± 8.0 years	500 mg/day for 4 weeks, followed or preceded by placebo; 8 week washout period	No significant change in pro‐inflammatory cytokines or antioxidant biomarkers	PBMC Nrf2 and NF‐*κ*B mRNA expression did not change significantly	Small exploratory crossover study; no PPAR biomarker; no plasma resveratrol or metabolite concentration data; a negative biomarker result may reflect dose, exposure, tissue selection, timing, or biological inactivity	[62]
Curcumin formulations: AOV curcumin extract, AOV curcumin extract plus piperine, Longvida, and NovaSOL	Healthy adult males; open‐label crossover pharmacokinetic study; *n* = 9	AOV curcumin extract: 600 mg extract containing 570 mg curcuminoids; high‐dose AOV: 2400 mg extract containing 2280 mg curcuminoids; high‐dose AOV plus piperine: 2280 mg curcuminoids plus 20 mg piperine; Longvida: 525 mg curcumin; NovaSOL: 576 mg curcuminoids; single‐dose administration.	NovaSOL produced the highest unconjugated and conjugated curcumin exposure among the tested formulations. Piperine did not increase systemic curcumin exposure in this study. No therapeutic efficacy outcome was evaluated.	No PPAR or NF‐*κ*B biomarker was measured. Unconjugated curcumin remained below the 2 nM lower limit of quantification after AOV, high‐dose AOV, AOV plus piperine, and Longvida. NovaSOL produced measurable unconjugated curcumin in all participants, with a median C_max_ of 14.9 nM (range, 6.7–38.4 nM) at 30 min.	Healthy‐volunteer PK study, not a patient efficacy trial; very small sample; plasma exposure was predominantly conjugated curcuminoids; free parent‐compound exposure remained substantially below common in vitro concentrations.	[57]

*Note:* This table includes human studies with either pathway‐relevant biomarkers or high quality pharmacokinetic evidence relevant to clinical translation. Increased or decreased PBMC expression of *PPARG*, *PPARA*, Nrf2, or NF‐*κ*B does not establish direct receptor binding, tissue‐specific PPAR activation, or PPAR‐dependent NF‐*κ*B transrepression. Curcumin results distinguish unconjugated parent compound from total curcuminoids measured after enzymatic hydrolysis of conjugates; these analytes should not be interpreted as interchangeable measures of biologically active exposure.

Abbreviations: AOV, commercially available curcumin extract formulation; C_max,_ maximum observed plasma concentration; HDL, high‐density lipoprotein; NF‐*κ*B, nuclear factor‐kappa B; Nrf2, nuclear factor erythroid 2 related factor 2; PBMC, peripheral blood mononuclear cell; PPAR, peroxisome proliferator‐activated receptor; sCD163, soluble cluster of differentiation 163; SIRT1, sirtuin 1; sTWEAK, soluble TNF like weak inducer of apoptosis.

Resveratrol provides the clearest example of both the promise and limitations of the available clinical literature. In a randomized, double‐blind, placebo‐controlled trial involving 56 patients with Type 2 diabetes mellitus and coronary heart disease, 500 mg/day of resveratrol for 4 weeks increased *PPARG* and *SIRT1* expression in peripheral blood mononuclear cells and was associated with favorable changes in selected glycemic and lipid‐related outcomes [60]. However, a separate double‐blind trial in 71 patients with Type 2 diabetes mellitus found that 1000 mg/day of trans‐resveratrol for 8 weeks did not significantly modify *PPARA* expression, despite changes in selected senescence‐related markers [61]. Similarly, a randomized, double‐blind, placebo‐controlled crossover study in 20 nondialyzed chronic‐kidney‐disease patients found that 500 mg/day of resveratrol for 4 weeks did not significantly alter NF‐*κ*B or Nrf2 expression, inflammatory biomarkers, or oxidative stress markers [62]. Collectively, these findings indicate that human biomarker effects are context‐dependent and do not yet establish a reproducible clinical PPAR–NF‐*κ*B mechanism.

Compared with phytochemicals, approved synthetic PPAR agonists have defined indications, regulated manufacturing standards, established pharmacokinetic profiles, and better‐characterized efficacy. Thiazolidinediones, particularly pioglitazone, are clinically effective insulin sensitizers through PPAR‐*γ* activation, whereas fibrates are used primarily to lower triglycerides through PPAR‐*α* activation. Their use is nevertheless constrained by adverse effects and monitoring requirements. PPAR‐*γ* agonists can cause weight gain, fluid retention, edema, heart failure exacerbation, and increased fracture risk, whereas fibrates require attention to hepatic effects, gallstone risk, myopathy, and potential rhabdomyolysis, particularly when combined with statins [63].

Natural phytochemicals should not be presumed safer simply because they are plant derived. Their safety profile may be influenced by dose, duration, formulation, coadministered drugs, active metabolites, botanical source, and batch‐to‐batch variation. Existing human studies are generally small, short, and heterogeneous in formulation and outcome selection; thus, they are not adequate to establish long‐term safety, clinically meaningful herb‐drug interaction risk, or comparative effectiveness against approved PPAR‐targeting medicines. Future trials should integrate validated pharmacokinetic sampling with mechanistic biomarkers. At minimum, reports should specify the biological matrix, analytical platform, lower and upper limits of quantification, parent compound and metabolite measurements, timing of sampling, and whether analytes represent free or total concentrations. Without these details, a negative biomarker result may reflect inadequate exposure, an insensitive assay, an inappropriate sampling window, or absence of biological activity.

### 6.3. Biomarker‐Driven Trials and Research Priorities

Future development of phytochemical PPAR modulators should follow a predefined translational sequence linking compound identity and formulation to measured exposure, PPAR target engagement, NF‐*κ*B pathway modulation, and clinically relevant outcomes. Randomized trials should not rely solely on nonspecific endpoints, such as body weight, lipid profile, C‐reactive protein, or individual circulating cytokines. Instead, they should determine whether the parent phytochemical or an active metabolite reaches a biologically relevant concentration and modulates the intended PPAR–NF‐*κ*B pathway in human participants.

Target engagement biomarkers should be selected according to the PPAR isoform, disease indication, and sampled tissue. PPAR‐*α* oriented studies may assess *CPT1A*, *HMGCS2*, *ACSL1*, *FABP1*, and *ACOX1* in liver relevant or metabolically appropriate samples. For PPAR‐*γ* oriented candidates, *PPARG*, *CD36*, *FABP4*, adiponectin, and macrophage‐polarization signatures may be assessed in peripheral blood mononuclear cells, adipose tissue, or other disease‐relevant specimens. NF‐*κ*B pathway assessment should extend beyond a single cytokine and may include phospho‐p65/RelA, p65 nuclear localization, IKK/I*κ*B‐*α* phosphorylation, and transcriptional changes in *NFKBIA*, *TNFAIP3*, *TNF*, *IL1B*, *IL6*, *CCL2*, and *PTGS2*.

Biomarker interpretation should be integrated with validated pharmacokinetic measurements of free parent compound and major metabolites. Reports should specify the biological matrix, analytical platform, calibration range, lower and upper limits of quantification, analyte stability, sampling time, and whether concentrations represent unconjugated or total compound. Without these data, a negative biomarker result may reflect inadequate exposure or assay limitations rather than absence of biological activity.

Preclinical prioritization should favor purified compounds with direct binding, reporter transactivation, cofactor recruitment, or receptor dependency evidence. Antagonist experiments provide useful initial support, but gene silencing, CRISPR‐based editing, knockout models, rescue experiments, primary human cells, organoids, and humanized PPAR models offer stronger mechanistic validation. Species‐specific differences in PPAR‐*α* regulation, ligand response, metabolism, and downstream gene expression should be considered before extrapolating rodent efficacy to humans [64, 65]. Future trials should therefore use chemically characterized, batch‐standardized preparations; prespecify exposure and pathway biomarkers; include suitable placebo or active comparators; and prospectively monitor hepatic, renal, metabolic, hematological, and drug interaction safety outcomes.

## 7. Conclusion

This review indicates that phytochemicals can be stratified according to the strength and type of evidence supporting PPAR modulation and NF‐*κ*B related effects. A small group of compounds including amorfrutins, falcarindiol, honokiol, gypenoside XLIX, and linalool has direct PPAR binding and/or receptor‐functional evidence; however, direct PPAR‐dependent NF‐*κ*B suppression has not been demonstrated uniformly across these compounds. Several others, including naringin, naringenin, kaempferol, ursolic acid, oleanolic acid, puerarin, and *β*‐caryophyllene, show functional PPAR involvement or PPAR‐associated signaling in specific models alongside anti‐inflammatory or NF‐*κ*B‐related effects but lack definitive direct ligand evidence or a consistently demonstrated causal PPAR–NF‐*κ*B pathway. Across all groups, systemic exposure to free parent compounds after oral dosing is typically in the low nanomolar to submicromolar range, whereas most cellular PPAR and NF‐*κ*B experiments use 5–50 *μ*M, creating a persistent exposure potency gap that formulation strategies have not yet resolved in terms of target engagement. Future work should therefore distinguish direct PPAR ligand activity from receptor dependency and parallel pathway observations, couple formulation development with pharmacokinetic and target‐engagement endpoints, and design clinical trials around exposure ranges that are achievable in humans. Within these constraints, selected phytochemicals remain useful tools for probing PPAR biology and inflammation, but none should be described as a clinically validated selective PPAR modulator and claims of direct PPAR‐mediated NF‐*κ*B regulation should be reserved for systems in which receptor dependency has been experimentally demonstrated.

## Author Contributions

Conceptualization: Sabi Ur Rehman and Fazal Ur Rehman. Data collection: Sabi Ur Rehman, Amir Zeb, and Saleha Ashfaq. Data analysis: Sabi Ur Rehman and Fazal Ur Rehman. Writing and editing: Sabi Ur Rehman, Amir Zeb, Shakir Ullah Khan, and Shafi Ullah Khan. Proofreading: Waheed Ullah Hafiz, Saleha Ashfaq, and Shafi Ullah Khan. Correspondence: Waheed Ullah Hafiz.

## Funding

No funding was received for this manuscript.

## Disclosure

The initial draft of this manuscript was prepared by the authors. All authors critically reviewed and verified the final manuscript and take full responsibility for its accuracy, integrity, references, and conclusions.

## Conflicts of Interest

The authors declare no conflict of interest.

## Supporting information


**Supporting Information** Additional supporting information can be found online in the Supporting Information section. Table S1 Evidence supporting the involvement of natural compounds in PPAR signaling and inflammatory regulation. Table S2: Selected quantitative pharmacokinetic and formulation evidence relevant to clinical translation of phytochemical PPAR modulators.

## Data Availability

Data sharing is not applicable to this article as no new data were created or analyzed.
