# Supplementary material for: Phytochemicals at the PPAR–NF‐κB Interface: A Structured Narrative Review of Receptor Pharmacology, Inflammatory Signaling, and Translational Barriers
Source: PPAR Res. 2026 Oct 29;2026:9577380. doi: 10.1155/ppar/9577380 (PMC13718453; doi:10.1155/ppar/9577380)
Supplement: Supplementary file 1 [file PPAR-2026-9577380-s001.docx]

**Supplementary Table S1: Evidence supporting the involvement of natural compounds in PPAR signaling and inflammatory regulation**

| **Phytochemical** | **Chemical class** | **Representative natural occurrence / botanical source** | **Experimental model(s)** | **PPAR-related finding** | **NF-**κ**B-related / anti-inflammatory finding** | **PPAR-dependency or direct receptor evidence** | **Final evidence classification** | **Interpretation and principal limitation** | **Key primary source(s) for EndNote import** |
| --- | --- | --- | --- | --- | --- | --- | --- | --- | --- |
| Naringin | Flavanone glycoside | Citrus fruits, particularly grapefruit and related species | DSS-induced colitis mice; inflammatory cellular models; spinal cord injury model | Enhanced PPAR-γ associated signaling and expression | Reduced NF-κB activation, inflammatory cytokines, oxidative stress, and tissue injury | PPAR-γ involvement supported by inhibitor/siRNA studies; no direct ligand-binding evidence | PPAR-γ associated anti-inflammatory candidate | Functional PPAR-γ involvement does not establish direct agonism | [66, 67] |
| Naringenin | Flavanone aglycone | Citrus fruits, particularly grapefruit and related species | Primary rat hepatocytes and human hepatocytes; high-glucose H9c2 cardiomyocytes; streptozotocin-induced diabetic mice and cardiomyocytes | Activated PPAR-α and PPAR-γ ligand-binding-domain reporters and the PPRE in hepatocyte studies; PPAR signaling was also implicated in cardiomyocyte protection | Reduced NF-κB expression, nuclear NF-κB activity, pro-inflammatory cytokines, and oxidative injury in diabetic cardiomyopathy | Functional receptor-reporter evidence is available; no direct receptor-binding affinity was reported, and the NF-κB study did not test PPAR dependency | Functional PPAR-α/γ activator with independent anti-inflammatory activity | Existing evidence supports PPAR activation and separate anti-inflammatory observations, but does not establish naringenin as a validated dual PPAR–NF-κB modulator or a direct PPAR ligand | [68-70] |
| Kaempferol | Flavonol | Widely distributed in plant foods, including tea, onions, vegetables and berries | PPRE-luciferase reporter and SW480 cell assays; AGE-stimulated cells; aged rat kidney and gingival-tissue models | Increased PPRE-mediated PPAR-γ transactivation and OCTN2 expression; GW9662 inhibited kaempferol-associated reporter and OCTN2 effects | Suppresses NF-κB activation and inflammatory mediators | Functional PPAR-γ reporter and antagonist evidence is available; direct receptor-binding evidence is limited | PPAR-γ–associate compound with independent NF-κB inhibition | A causal PPAR-γ mediated NF-κB inhibitory pathway has not been demonstrated; PPAR-γ activation and NF-κB inhibition are supported but not mechanistically linked | [71-73] |
| Ursolic acid | Pentacyclic triterpene | Apple peel, holy basil | Rat cerebral ischemia/reperfusion model; LPS/IFN-γ-activated BV2 microglia | Promotes PPAR-γ dependent MMP2 transcription through PPRE mechanisms | Reduces, MMP2/MMP9 associated inflammatory injury and pro-inflammatory microglial response | BADGE attenuated PPAR-γ associated effects in ischemia and microglial models; the microglial study demonstrates PPAR-γ dependent MMP2 regulation at the PPRE level | PPAR-γ dependent anti-inflammatory candidate with PPRE-level mechanistic support | This is stronger than simple PPAR-γ expression evidence. Nevertheless, a PPRE in a downstream target gene does not establish direct ursolic-acid binding to PPAR-γ, and the cited studies do not directly establish PPAR-γ–dependent NF-κB inhibition. | [74, 75] |
| Oleanolic acid | Pentacyclic triterpene | Olive fruit and leaves; medicinal and aromatic plants; fruit peels | DSS-induced acute colitis in C57BL/6 mice; colonic epithelial and inflammatory-cell studies | Maintains PPAR-γ expression by inhibiting ubiquitin-mediated proteasomal degradation and stabilizing PPAR-γ protein | Suppresses NF-κB pathway activation and cytokines | PPAR-γ dependence demonstrated pharmacologically | PPAR-γ dependent anti-inflammatory candidate via receptor stabilization | The study strongly supports PPAR-γ dependent protection in DSS colitis. However, it indicates post-translational PPAR-γ stabilization rather than direct receptor binding or classical quantitative PPAR-γ agonism | [76] |
| Puerarin | Isoflavone C-glycoside | *Pueraria lobata* root | PPRE-luciferase reporter and SW480 cell assays; myocardial ischemia/reperfusion, vascular, and inflammatory models | Increased PPRE-mediated transcriptional activity and OCTN2 expression; GW9662 inhibited puerarin-associated PPAR-γ reporter activity | Reduced NF-κB activation, inflammatory cytokines, and NLRP3-inflammasome activity in myocardial ischemia/reperfusion studies | Functional PPAR-γ reporter and GW9662 evidence is available; direct receptor-binding evidence was not identified | Functional PPAR-γ activator with independently supported NF-κB inhibition | Existing evidence does not establish one causal PPAR-γ –NF-κB pathway. In myocardial ischemia/reperfusion, NF-κB-related protection was linked to SIRT1/NF-κB/NLRP3 signaling rather than demonstrated PPAR-γ dependency | [71, 77] |
| β-Caryophyllene | Bicyclic sesquiterpene | Essential oils of aromatic plants | DSS-induced colitis in mice; Aβ _25-35_ stimulated human HMC3 microglial cells | PPAR-γ participates in anti-inflammatory responses in colitis and microglial models | Reduced NF-κB activation, inflammatory cytokines, colonic injury, and microglial inflammatory response | GW9662 reduced protective or anti-inflammatory effects; the CB2 antagonist AM630 also reversed protective responses in colitis experiments | PPAR-γ -linked anti-inflammatory candidate with CB2–PPAR-γ cross-talk | Both CB2 and PPAR-γ contribute to the anti-inflammatory phenotype. Because β-caryophyllene is a CB2 agonist and both pathways are implicated, it should not be characterized as a selective direct PPAR-γ agonist or SPPARM | [78, 79] |
| Linalool | Monoterpene alcohol | Essential oils of lavender, coriander, sweet basil, bergamot and other aromatic plants | PPAR-α reporter-gene and TR-FRET assays; hepatocytes; PPAR-α knockdown cells; wild type and PPAR-α deficient mice; UVB exposed human dermal fibroblasts; LPS-stimulated BV2 microglia | Direct PPAR-α ligand activity was demonstrated by transactivation and TR-FRET coactivatorrecruitment assays; metabolic effects were lost after PPAR-α knockdown and in PPAR-α deficient mice | Reduced NF-κB/IκB-α related signaling in UVB-stimulated fibroblasts and inflammatory signaling in BV2 microglia | Yes: for PPAR-α: direct ligand and receptor-dependency evidence. No PPAR-α dependency evidence was reported for the NF-κB findings | Direct PPAR-α ligand with separately reported NF-κB inhibition | Linalool has the strongest direct PPAR-α evidence among the Section 5.4 candidates. Its anti-inflammatory effects were not demonstrated to be PPAR-α dependent; in BV2 microglia, Nrf2/HO-1 was implicated | [80-82] |

**Supplementary Table S1 footnote:**

**Abbreviations:** AGE, advanced glycation end product; Aβ _25-35_, amyloid-β peptide fragment 25-35; BADGE, bisphenol A diglycidyl ether; CB2, cannabinoid receptor type 2; DSS, dextran sulfate sodium; IκB-α, inhibitor of nuclear factor-kappa B alpha; IKK, IκB kinase; LBD, ligand-binding domain; LPS, lipopolysaccharide; MMP, matrix metalloproteinase; NF-κB, nuclear factor-kappa B; OCTN2, organic cation/carnitine transporter 2; PPAR, peroxisome proliferator-activated receptor; PPRE, peroxisome proliferator response element; TR-FRET, time-resolved fluorescence resonance energy transfer.

**Representative natural occurrence / botanical source:** This column provides selected, commonly reported natural sources for chemical and botanical context only. It does not indicate the provenance, extraction method, plant part, purity, formulation, or commercial supplier of the compound used in each cited study. Where a compound is widely distributed among plant taxa, representative rather than exhaustive sources are listed.

**Interpretive framework:** “Direct PPAR ligand” requires direct binding, ligand-binding-domain engagement, coactivator recruitment, and/or robust receptor-dependency evidence. “PPAR-involved” or “PPAR-dependent” indicates that the phenotype was reduced or reversed by pharmacological antagonism, knockdown, or related functional experiments; these findings do not independently establish direct binding. “Separately reported NF-κB inhibition” indicates that PPAR and NF-κB outcomes were reported in different studies or models without demonstrating a causal PPAR-to-NF-κB pathway. None of the phytochemicals in this supplementary table should be characterized as a clinically validated SPPARM.

**Supplementary Table S2.** Selected quantitative pharmacokinetic and formulation evidence relevant to clinical translation of phytochemical PPAR modulators

| **Phytochemical / formulation** | **Species, study design, and route** | **Dose** | **Analyte and analytical approach** | **Key pharmacokinetic result** | **Formulation-related finding** | **Translational interpretation and limitation** | **Primary reference** |
| --- | --- | --- | --- | --- | --- | --- | --- |
| Curcumin formulations**:** AOV curcumin extract, AOV curcumin extract + piperine, Longvida®, and NovaSOL® | Healthy adult males; independent crossover pharmacokinetic study; n=9; oral single-dose administration | AOV curcumin extract: 600 mg (570 mg curcuminoids); high-dose AOV: 2400 mg (2280 mg curcuminoids); high-dose AOV + piperine: 2400 mg curcuminoids plus 20 mg piperine; Longvida®: 525 mg; NovaSOL®: formulation-specific curcuminoid dose as evaluated in the study. | Unconjugated curcumin was measured directly without enzymatic hydrolysis, while total curcuminoids were assessed after enzymatic deconjugation. Plasma quantification used LC-MS/MS-based analysis. | Plasma unconjugated curcumin remained below the limit of quantification (2 nM) for most formulations, including high-dose AOV and piperine-containing formulations. NovaSOL® produced measurable unconjugated curcumin exposure (median Cmax 14.9 nM; range 6.7–38.4 nM). Total curcumin concentrations were substantially higher because of circulating conjugates. | NovaSOL® produced the highest systemic curcumin exposure, whereas piperine addition did not increase systemic uptake. | Human PK evidence demonstrates formulation-dependent exposure differences. However, enhanced total curcuminoid exposure mainly reflects conjugated metabolites and should not be interpreted as increased free curcumin availability, PPAR target engagement, or biological efficacy. | [46] |
| Trans-resveratrol | Healthy volunteers; phase I dose-escalation pharmacokinetic study; n=40 (10 participants per dose group); oral single-dose administration. | 0.5, 1.0, 2.5, or 5.0 g resveratrol administered as immediate-release 500 mg caplets. | Resveratrol and metabolites were identified in plasma and urine by HPLC-tandem mass spectrometry and quantified by HPLC-UV. Metabolite concentrations were expressed as resveratrol equivalents because authentic standards were unavailable for all metabolites. | At the highest dose (5 g), parent resveratrol reached a mean Cmax of 538.8 ng/mL (2.4 µM) with Tmax of 1.5 h and AUC∞ of 1319 ng·h/mL. Resveratrol-3-sulfate and glucuronide metabolites showed substantially greater systemic exposure, with AUC values up to 23-fold higher than parent resveratrol. | Oral administration resulted in rapid absorption but extensive phase II metabolism, with sulfate and glucuronide conjugates representing the predominant circulating species. | Human PK evidence indicates limited systemic exposure to parent resveratrol after oral dosing. Although conjugated metabolites achieve higher circulating concentrations, their contribution to PPAR/NF-κB modulation remains incompletely defined. | [83] |
| Honokiol/magnolol lecithin based mixed polymeric micelles: lbMPMs[NaDOC] | Male Sprague Dawley rats (8-10 weeks old); pharmacokinetic study comparing optimized micellar formulation with free honokiol/magnolol solution and lbMPMs[PP123]; single-dose intravenous and oral administration | Intravenous administration: 20 mg/kg of honokiol/magnolol-loaded lbMPMs[NaDOC], lbMPMs[PP123], or free compound solution. Oral administration: 40 mg/kg of the corresponding formulations. | Plasma honokiol and magnolol concentrations were quantified by UPLC/MS/MS. Pharmacokinetic parameters were determined using non-compartmental analysis. | lbMPMs[NaDOC] improved systemic exposure compared with free compounds. Following oral administration, honokiol absolute bioavailability was 4.8%, whereas magnolol showed an absolute bioavailability of 20.1% and a 2.9-fold increase in relative bioavailability compared with free magnolol. | The optimized lbMPMs[NaDOC] formulation (honokiol/magnolol:lecithin:NaDOC = 6:2:5) improved solubility and oral bioavailability. The formulation showed high encapsulation efficiency (>90%) and enhanced exposure compared with lbMPMs[PP123]. | Preclinical rat PK evidence demonstrates formulation-dependent improvement in exposure. However, increased systemic availability does not establish human bioavailability, clinical efficacy, PPAR target engagement, or NF-κB pathway modulation. | [47] |

**Supplementary Table S2 footnote**: **Abbreviations:** AOV, commercially available curcumin extract formulation; *AUC*_∞_, area under the plasma concentration time curve extrapolated to infinity; C_max_, maximum observed plasma concentration; HPLC, high-performance liquid chromatography; lbMPMs, lecithin based mixed polymeric micelles; NaDOC, sodium deoxycholate; NF-κB, nuclear factor-kappa B; PPAR, peroxisome proliferator-activated receptor; $T_{\max}$, time to maximum observed plasma concentration.

**Interpretive framework:** This table includes selected primary studies reporting human or animal pharmacokinetic parameters, formulation-dependent exposure, or absolute/relative bioavailability. Values from different species, doses, routes, formulations, matrices, assay platforms, and analytes must not be interpreted as directly comparable. Free parent compound, total compound after enzymatic deconjugation, and glucuronide/sulfate metabolites are analytically distinct measures that may have different pharmacological relevance. Improved Cmax, AUC, or bioavailability indicates enhanced systemic exposure but does not by itself establish increased free active compound availability, PPAR target engagement, PPAR-dependent NF-κB modulation, clinical efficacy, or long-term safety. “Not reported in the abstract” means that the value was not available in the audited PubMed or publisher record and should not be inferred, estimated, or imputed.
